# Blunt head impact causes a temperature rise in the brain

**DOI:** 10.1098/rsos.220890

**Published:** 2022-11-30

**Authors:** Amit Madhukar, Martin Ostoja-Starzewski

**Affiliations:** Department of Mechanical Science and Engineering, Beckman Institute, and Institute for Condensed Matter Theory, University of Illinois at Urbana-Champaign, Champaign, IL 61801, USA

**Keywords:** brain injury, thermoelasticity, brain thermomechanics

## Abstract

A magnetic resonance imaging-based finite-element model is employed to assess the temperature in the human brain due to blunt head trauma. The model is based on a coupled thermoelasticity under small strain and Fourier or Maxwell–Cattaneo heat conduction assumptions, accompanied by a standard coupling of thermal fields to mechanics. It is found that mechanical impacts on the forehead cause a temperature rise of up to 0.3°C above the reference homogeneous temperature field.

## 1. Background and motivation

The last two decades have seen progressive development of mechanics models of traumatic brain injury (TBI) (see e.g. [1,2]). Modern models are based on magnetic resonance imaging (MRI) and, providing a sufficiently fine spatial resolution, detailed stress and displacement wave patterns can be obtained [3,4]. Most recently, additional aspects of TBIs have been uncovered due to the introduction of magnetic resonance elastography (MRE) [5]. Most importantly this allows models to accurately capture heterogeneities in white matter tracts of the cerebrum. However, mechanics models of TBI are typically based on the isothermal assumption, effectively neglecting the temperature fields in the brain and any coupling of thermal fields to mechanics. A recent study on rapid heating of brain tissue through electromagnetic radiation found that such temperature rise produced stresses that are comparable to those seen in blast and ballistic loading [6]. This report shows that admitting a thermoelastic coupling in the brain tissue results in a temperature rise. This coupling is analogous to that present in the constitutive modelling of rubber: part of the mechanical energy of a wave gets irreversibly converted into heat.

### 1.1. Model formulation

The deformation of dynamically stressed tissues is neither isothermal nor adiabatic. Hence, the strain tensor and temperature are two independent state variables*.* The following governing equations are used for the simplest model of the coupled thermoelasticity of brain tissues. First, we have the usual balance laws of mechanics,

1.1 $$\rho \frac{\partial^{2}\mathrm{>u}}{\partial t^{2}}\;=\text{div}(C:\varepsilon (u)+m\theta )+\rho b,$$

where u, ε, b  and θ are the displacement, strain, body force and temperature fields, respectively. *ρ* is the mass density, C is the fourth-rank stiffness tensor and m is a second-rank tensor representing the coupling term between mechanical and thermal fields. If we assume hyperbolic-type heat conduction starting from the Maxwell–Cattaneo flux law, we also have

1.2 $$\rho C_{p}\left(\frac{\partial \theta }{\partial t}+\tau_{0}\frac{\partial^{2}\theta }{\partial t^{2}}\right)=\text{div(}\kappa \cdot \nabla \theta \text{)}+\theta_{0}m:\left(\frac{\partial }{\partial t}\nabla u+\tau_{0}\frac{\partial^{2}}{\partial t^{2}}\nabla u\right),$$

where $\tau_{0}$ is the thermal relaxation time, κ is the second-rank conductivity tensor, and *θ*_0_ is the initial temperature. The material tensors C**,** κ and m can be simplified under the assumptions of isotropy as

1.3 C=λI⊗I+2μ1,

1.4 κ=κI,

1.5 andm=mI=−(3λ+2μ)αI,

where λ and μ are the Lamé constants, κ is the thermal diffusivity and α is the coefficient of thermal expansion. I and 1 are the usual second- and fourth-order identity tensors, respectively. *c*_*p*_ is the specific heat at constant pressure. The usual parabolic case of heat conduction can be recovered by setting thermal relaxation time $\tau_{0}$ to zero [7].

The governing equations are solved using the explicit central-difference integration rule in space and forward-difference in time with a lumped mass matrix as described below. Since both the forward-difference and central-difference integrations are explicit, the heat transfer and mechanical solutions are obtained simultaneously by an explicit coupling. The formulation is based on the fully coupled thermal-stress analysis in ABAQUS/Explicit where the matrix system can be represented as

1.6 $$\left[\begin{array}{cc} K_{uu} & K_{u\theta }\\ K_{\theta u} & K_{\theta \theta } \end{array}\right]\left\{\begin{array}{c} \Delta u\\ \Delta \theta \end{array}\right\}=\;\left\{\begin{array}{c} R_{u}\\ R_{\theta } \end{array}\right\},$$

where Δu and Δθ are the corrections to the incremental displacement and temperature, respectively; $K_{ij}$ are the submatrix of the fully coupled Jacobian matrix and $R_{u}$ and $R_{\theta }$ are the mechanical and thermal residual vectors, respectively.

We use the explicit forward difference-time integration scheme to perform the time-stepping,

1.7 $$\theta_{\left(i+1\right)}^{N}=\;\theta_{\left(i\right)}^{N}+\;\Delta t_{\left(i+1\right)}{\dot{\theta }}_{\left(i\right)}^{N},$$

where the notation $\theta_{\left(i\right)}^{N}$ corresponds to the temperature at node *N* at time-step *i*. The value of ${\dot{\theta }}_{\left(i\right)}^{N}$ is computed as ${\dot{\theta }}_{\left(i\right)}^{N}={\left(C^{NJ}\right)}^{-1}(P_{\;(i)}^{J}-\;F_{\;(i)}^{J})$, where $C^{NJ}$ is the lumped capacitance matrix, $P_{\;(i)}^{J}$ is the applied nodal source vector and $F_{\;(i)}^{\;J}$ is the internal flux vector.

**Figure 1.**
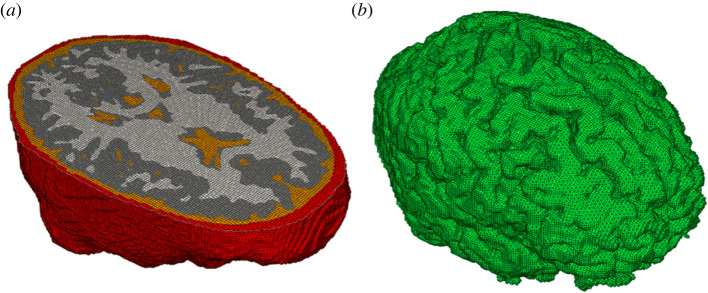
Finite-element model [3–5]. (*a*) Transverse (axial) section depicting different element types. (*b*) The outer surface of the cerebrum.

Our finite-element (FE) head model [4,5] is segmented into standard tissue types through image segmentation—skull, cerebrospinal fluid (CSF), white matter and grey matter. The resolution of each voxel is taken directly from the imaging resolution. The final model has roughly 1 million elements with 1.33 × 1.33 × 1.30 mm hexahedral elements (figure 1). On the length scales of single FEs, mechanical properties of the brain's grey and white matter are assumed to be linearly isotropic and homogeneous. Brain behaviour in shear is characterized by the standard linear viscoelastic model. The mechanical properties of these tissues are presented in table 1. The mechanical properties of the white and grey matter tissues were taken from the model by Zhang *et al*. [8] which in turn are based on the experiments performed by Fallenstein *et al*. [9] and Shuck & Advani [10]. The thermal properties of the tissues are presented in table 2.

**Table 1.** Mechanical properties of different tissues used in the FE model [3,4].

tissue	mass density (kg m^−3^)	bulk modulus *K* (Pa)	short-term shear modulus *G*_0_ (Pa)	long-term shear modulus *G*_∞_ (Pa)	decay factor β (s^−1^)
skull	2070	3.61 × 10^9^	2.7 × 10^9^		n.a.
grey matter	1040	2.19 × 10^9^	3.4 × 10^4^	6.4 × 10^3^	400
white matter	1040	2.19 × 10^9^	4.1 × 10^4^	7.8 × 10^3^	400
CSF (solid incompressible)	1004	2.19 × 10^7^	5.0 × 10^4^		n.a.

**Table 2.** Thermal properties of different tissues used in the FE model [11,12].

tissue	thermal conductivity (J °C^−1^ ms^−1^)	specific heat (J kg^−1^ °C^−1^)	expansion coefficient (°C^−1^)
skull	0.41	3930	8 × 10^−5^
grey matter	0.505	3930	8 × 10^−5^
white matter	0.505	3930	8 × 10^−5^
CSF	0.505	3930	8 × 10^−5^

The model has been previously validated for the case of pure mechanical response with good agreement with experimental studies [2–5]. In particular, we find that our linear model in [2,3] accurately captures the coup and counter-coup pressure response observed in the experiments performed by Nahum *et al.* [13]. Additionally, the model is also verified using tagged MRI and harmonic phase (HARP) imaging analysis techniques in [14], where displacement time history from head-drop experiments is compared with numerical results. While we have previously considered large-strain effects of brain tissues [5], the present study is limited to the small-strain assumption in order to reduce the model's complexity. While this assumption is generally not as valid as a nonlinear material model, we nevertheless use the linear model in an effort to reduce computational cost of this initial version of the model.

### 1.2. Loading parameters and initial conditions

**Figure 2.**
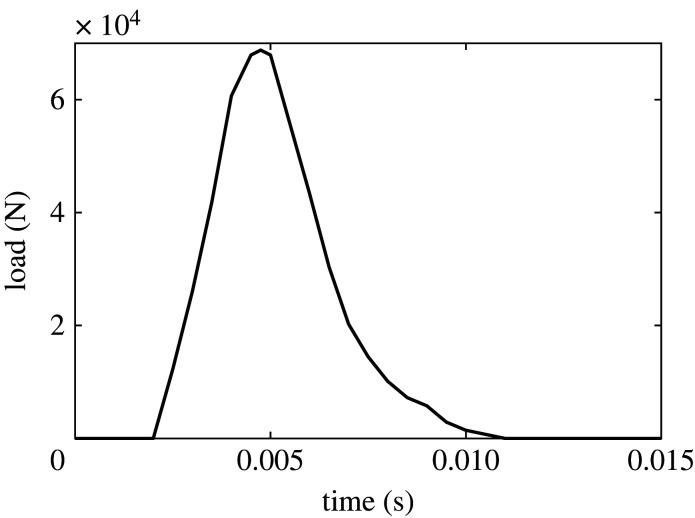
Impact loading time history.

Brain impact loading is based on the experiments in [13]. We scale the loading by a factor of 10 from these experiments in the simulations presented here (figure 2). The impact load is applied to the mid-frontal area in the anterior–posterior direction and the form of a distributed load. The head–neck junction is taken to be entirely free of constraints. The initial temperature of the entire model (throughout the brain) is taken to be 37°C.

To study the coup impact (to the forehead), we use a fully coupled thermal-stress analysis in ABAQUS/Explicit. The time incrementation parameters are automatically chosen to ensure the method's stability, with a small-strain assumption employed to limit the execution time. Mechanical and thermal contact properties are defined at the interfaces between each tissue type.

## 2. Results and discussion

**Figure 3.**
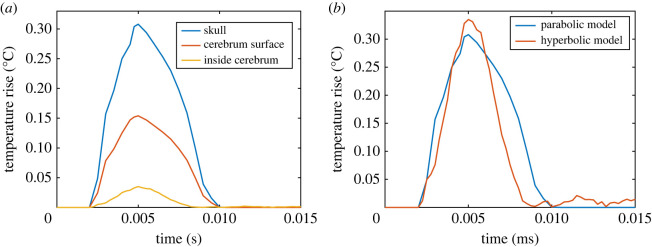
(*a*) Results for temperature rise history at three positions along the sagittal plane in line with the applied load. (*b*) Comparison between parabolic and hyperbolic models.

The impact load as defined above produces a temperature rise inside the brain, depicted in figure 3*a*. The temperature rise is measured for three positions along the sagittal plane directly in line with the applied distributed load. We find that the maximum temperature rise is roughly 0.3°C at the point directly on the skull at the impact point (blue curve). On the surface of the cerebrum and directly behind the applied load, the temperature rise is 0.15°C (red curve). Finally, inside the cerebrum (at a position roughly 30 mm behind the load application) the heat rise is reduced to 0.03°C (orange curve). In all cases, the heat rise is in phase with the applied loading. The simulation runtime for the parabolic model is roughly 55 h for a serial computation in ABAQUS/Explicit; a desktop computer with an Intel i5–10600k processor @ 4.8 GHz with 16GB RAM was used.

**Figure 4.**
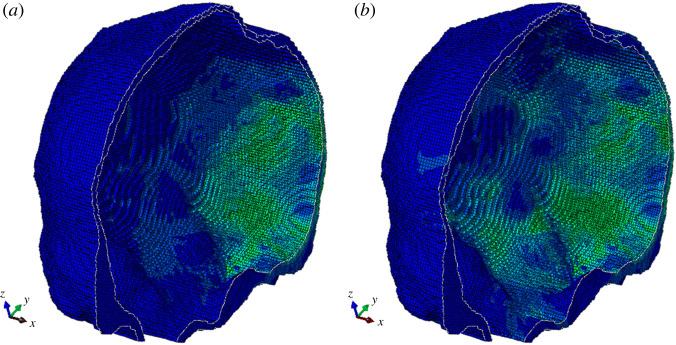
Temperature contours for sagittal plane of skull for 5 ms (*a*) and 7 ms (*b*).

Figure 3*b* depicts the comparison between the parabolic heat conduction model (based on the Fourier law, recovered from equations (1.1) and (1.2) by setting $\tau_{0}=0$) with the hyperbolic version (based on the Maxwell–Cattaneo law) using the telegraph equation implemented as a UMAT in ABAQUS. We use the thermal relaxation time $\tau_{0}=6.9\;s$. Figure 4 depicts the temperature contours for the sagittal plane of the skull for two time values. Table 3 presents the temperature rise at the same three positions along the sagittal plane for different values of $\tau_{0}$. Besides the small increase in temperature rise with rising $\tau_{0}$, it is found that there is little qualitative difference between the linear parabolic and hyperbolic heat conduction models. The simulation runtime for the hyperbolic model is roughly 250 h for serial computation.

**Table 3.** Temperature rise at three positions along the sagittal plane in line with the applied load for different values of $\tau_{0}$.

$\tau_{0}$	skull (°C)	cerebrum surface (°C)	inside cerebrum (°C)
0	0.308	0.154	0.035
3	0.312	0.167	0.038
6	0.329	0.173	0.041
6.9	0.335	0.175	0.041
9	0.339	0.181	0.048

The results obtained from the simplest possible thermoelastic model (equations (1.1)–(1.5)) strongly suggest that stress waves due to blunt impacts to the skull lead to temperature rise in the brain. Additionally, there is an indication that the plots in figure 3 would be altered to higher temperatures in the case of:

(a) local anisotropy of brain tissue;
(b) finer mesh resolution;
(c) nonlinear or fractional constitutive laws;
(d) finite deformations;
(e) stronger impacts, especially due to penetration of the brain by a bullet.

These effects will be considered in future works. Given that the brain tissue dies above 42°C, more in-depth research on the thermoelasticity under blunt head impacts is needed.

## Data Availability

The data are provided in electronic supplementary material [15].
